# Histopathological effects of modern topical sealants on the liver surface after hepatectomy: an experimental swine study

**DOI:** 10.1038/s41598-019-43694-6

**Published:** 2019-05-08

**Authors:** Hamidreza Fonouni, Elias Khajeh, Omid Ghamarnejad, Arash Kashfi, Emre Aydogdu, Ali Majlesara, Sara Mohammadi, Negin Gharabaghi, Lukas Konstantinidis, Thomas Longerich, Arianeb Mehrabi, Yakup Kulu

**Affiliations:** 1 Department of General, Visceral, and Transplantation Surgery, University of Heidelberg, Heidelberg, Germany; 2 Institute of Pathology, University of Heidelberg, Heidelberg, Germany

**Keywords:** Hepatocytes, Liver

## Abstract

The present study aimed to determine the impact of different sealant materials on histopathological changes to the liver surface after liver resection. Thirty-six landrace pigs underwent left anatomical hemihepatectomy and were assigned to a histopathological control group (HPC, n = 9) with no bleeding control, a clinically simulated control group (CSC, n = 9) with no sealant but bipolar cauterization and oversewing of the liver surface, and two treatment groups (n = 9 each) with a collagen-based sealant (CBS) or a fibrinogen-based sealant (FBS) on resection surface. After postoperative day 6, tissue samples were histologically examined. There were no significant differences in preoperative parameters between the groups. Fibrin production was higher in sealant groups compared with the HPC and CSC groups (both *p* < 0.001). Hepatocellular regeneration in sealant groups was higher than in both control groups. A significantly higher regeneration was seen in the FBS group. Use of sealants increased the degree of fibrin exudation at the resection plane. Increased hepatocellular necrosis was seen in the CBS group compared with the FBS group. The posthepatectomy hepatocellular regeneration rate was higher in the FBS group compared with the CBS group. Randomized studies are needed to assess the impact of sealants on posthepatectomy liver regeneration in the clinical setting.

## Introduction

Liver resection is the treatment of choice for patients with primary and secondary liver malignancies^1,2^. In recent decades, postoperative morbidity and mortality have decreased considerably thanks to a better understanding of liver anatomy and physiology, and remarkable developments in surgical instruments^3^. Nevertheless, the risk of resection-surface-related complications, such as bile leakage and hemorrhage, is still considerable^4,5^.

Dressing materials are used frequently to reduce these complications^6,7^. Dressing materials plug parenchymal and vascular microlesions by inducing local coagulation. The most commonly used dressing materials in hepatobiliary surgery are fibrin-impregnated and collagen-based fleeces^8^. However, the applications and advantages of, and histopathological reactions to, different sealants in hepatobiliary surgery have not been clearly defined.

Some experimental studies have compared the impact of sealants with other coagulation techniques and have demonstrated that sealants reduce necrosis^9^, fluid collection, and abscess formation^10^, and increase regeneration of the resection plane after liver resection^11^. However, to our knowledge, the postoperative histopathological effects of different sealant materials on the liver surface after hepatectomy have not been compared in detail so far. The aim of the present experimental study is to provide evidence-based data to determine the histopathological interactions of different sealants with the liver surface following liver resection. To do this, we assessed the postoperative histopathological effects of commonly used topical hemostats – a fibrin-based sealant (FBS) and a collagen-based sealant (CBS) – on resected liver surfaces in a swine model.

## Results

### Comparison of perioperative data

As shown in Table 1, all animals were hemodynamically stable and transhepatic flows were in the normal range intraoperatively before liver resection. There were no significant differences in preoperative data between the sealant groups and CSC or HPC groups. However, the relative bleeding time after resection was significantly different between sealant groups and the HPC group (*p* < 0.001). Post-hoc analysis revealed that the relative bleeding time in both FBS (*p* < 0.001) and CBS (*p* = 0.019) was significantly lower than in the HPC group. The relative bleeding time was also significantly lower in the FBS group compared with the CBS group (*p* = 0.022). Comparing sealant groups and the CSC group, the relative bleeding time was also significantly different between the groups (*p* = 0.029). However, according to post-hoc analysis there were no significant differences in relative bleeding time between FBS (*p* = 0.478) or CBS (*p* = 0.233) groups and the CSC group. There were also significant differences in absolute bleeding time between the FBS, CBS, and HPC groups (*p* < 0.001); absolute bleeding time was significantly lower in the FBS group than in the CBS (*p* = 0.002) and HPC groups (*p* < 0.001) according to post-hoc analysis. However, there were no significant differences between the FBS (*p* = 0.142) or CBS (*p* = 0.176) groups and the CSC group. Blood loss was significantly lower in both sealant groups than the HPC group (*p* < 0.001), but there was no significant difference between the sealant groups and the CSC group (*p* = 0.697). However, the difference between the FBS and CBS groups was not statistically significant. Furthermore, there were no significant differences in postoperative laboratory assessment results between the groups, including alanine transaminase (ALT), aspartate aminotransferase (AST), total bilirubin, albumin, and international normalized ratio (INR) (Table 1). No pigs died during the follow-up period.

**Table 1:** Comparison of the perioperative data between study groups.

Variables	HPC	CSC	FBS	CBS	p*	p**
Weight (kg)	29.8 ± 1.8	29.8 ± 2.1	30.0 ± 1.2	28.5 ± 2.1	0.161	0.196
Preoperative ALT (U/L)	45.7 ± 22.8	39.8 ± 7.0	39.7 ± 7.4	39.7 ± 5.3	0.591	0.999
Preoperative AST (U/L)	39.7 ± 8.1	38.3 ± 7.2	41.7 ± 8.2	45.2 ± 7.7	0.352	0.186
Preoperative total bilirubin (mg/dL)	0.21 ± 0.02	0.21 ± 0.01	0.20 ± 0.02	0.20 ± 0.01	0.382	0.243
Preoperative albumin (g/L)	32.1 ± 1.6	32.7 ± 1.8	33.2 ± 1.8	33.6 ± 1.8	0.186	0.575
Preoperative INR	0.94 ± 0.45	0.94 ± 0.03	0.94 ± 0.32	0.95 ± 0.03	0.997	0.991
Pre-resection heart rate	96.2 ± 14.8	92.7 ± 10.3	86.5 ± 6.5	100.8 ± 19.3	0.125	0.090
Pre-resection mean arterial pressure (mmHg)	59.5 ± 14.8	59.0 ± 10.9	62.5 ± 15	64.4 ± 8.5	0.729	0.620
Pre-resection hepatic arterial flow (ml/min)	0.1 ± 0.1	0.2 ± 0.1	0.2 ± 0.1	0.2 ± 0.1	0.068	0.999
Pre-resection portal vein flow (ml/min)	1.1 ± 0.3	1.1 ± 0.3	1.2 ± 0.4	1.1 ± 0.3	0.769	0.769
Relative bleeding time (sec)	307.7 ± 48.5	117.3 ± 15.8	70.0 ± 129.1	184.8 ± 70.0	**<0.001**	**0.029**
Absolute bleeding time (sec)	325.0 ± 18.4	167.3 ± 23.6	83.1 ± 125.2	246.3 ± 92.3	**<0.001**	**0.003**
Blood loss (g)	105.3 ± 74.7	18.2 ± 4.7	17.2 ± 47.8	8.0 ± 5.4	**<0.001**	0.697
Postoperative ALT (U/L)	43.5 ± 2.5	47.5 ± 7.6	46.7 ± 8.8	51.5 ± 8.7	0.084	0.441
Postoperative AST (U/L)	51.0 ± 9.1	47.3 ± 7.8	45.0 ± 6.6	48.5 ± 9.3	0.332	0.644
Postoperative total bilirubin (mg/dL)	0.49 ± 0.48	0.37 ± 0.22	0.26 ± 0.08	0.29 ± 0.10	0.202	0.278
Postoperative albumin (g/L)	31.4 ± 2.4	30.5 ± 1.8	31.3 ± 4.5	29.7 ± 6.3	0.691	0.763
Postoperative INR	0.99 ± 0.11	0.98 ± 0.09	0.94 ± 0.35	0.95 ± 0.36	0.931	0.956

CSC, clinically simulated control; HPC: histopathologic control; FBS: fibrin-based sealant; CBS: collagen-based sealant; ALT: alanine transaminase; AST: aspartate aminotransferase; INR: international normalized ratio.

*Comparison between HPC, FBS, and CBS groups.

**Comparison between CSC, FBS, and CBS groups.

### Histopathological evaluations

**Figure 1:**
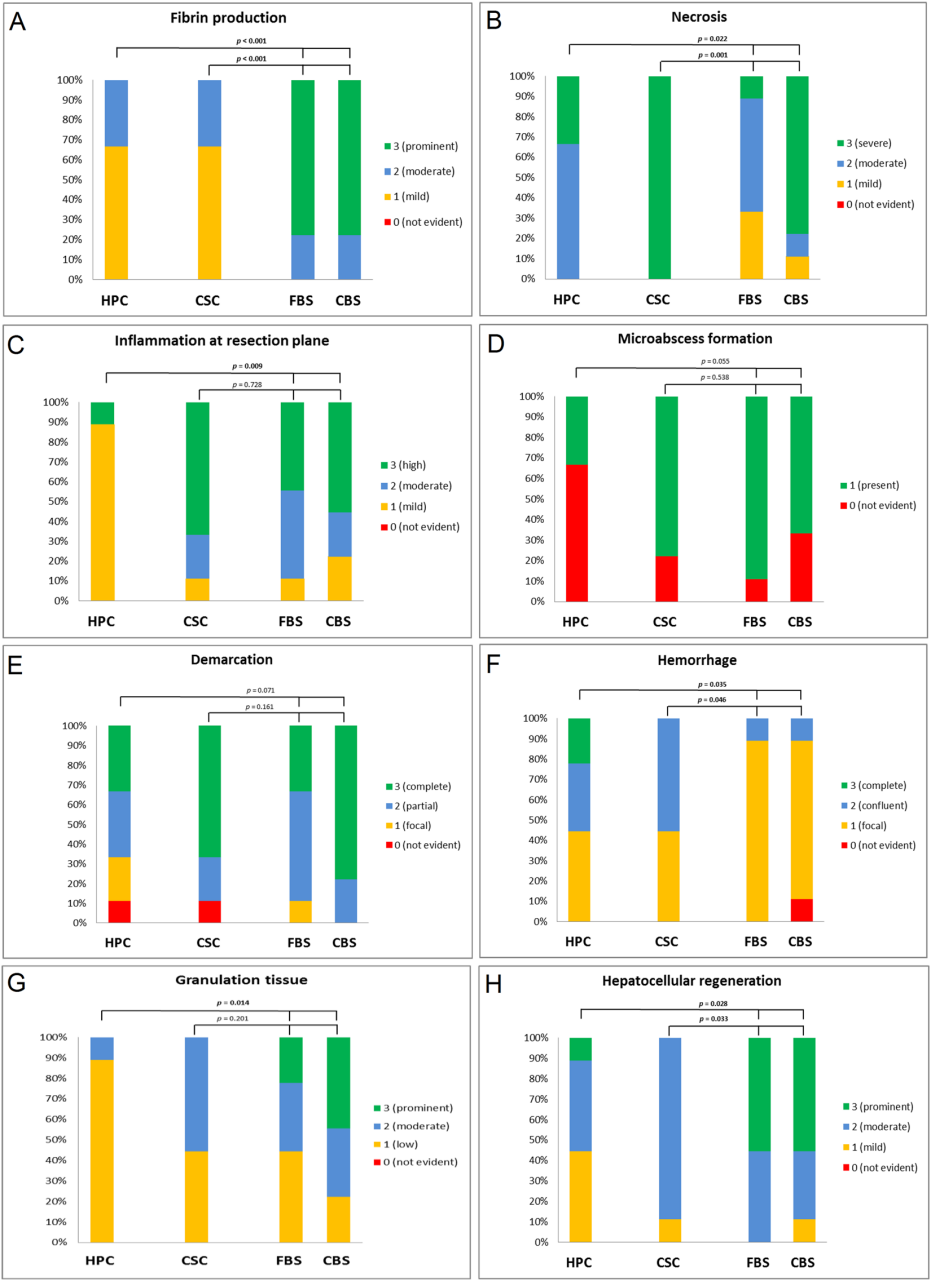
Histopathhological changes after hepatectomy in four different groups of HPC, CSC, FBS, and CBS (**A**) fibrin production, (**B**) necrosis, (**C**) inflammation at resection plane, (**D**) microabscess formation, (**e**) demarcation, (**F**) hemorrhage, (**G**) granulation tissue, and (**H**) hepatocellular regeneration*. P* values were calculated for comparison between intervention groups (FBS and CBS) and control groups (HPC and CSC) groups. HPC: histopathologic control; CSC, clinically simulated control; FBS: fibrin-based sealant; CBS: collagen-based sealant.

**Figure 2:**
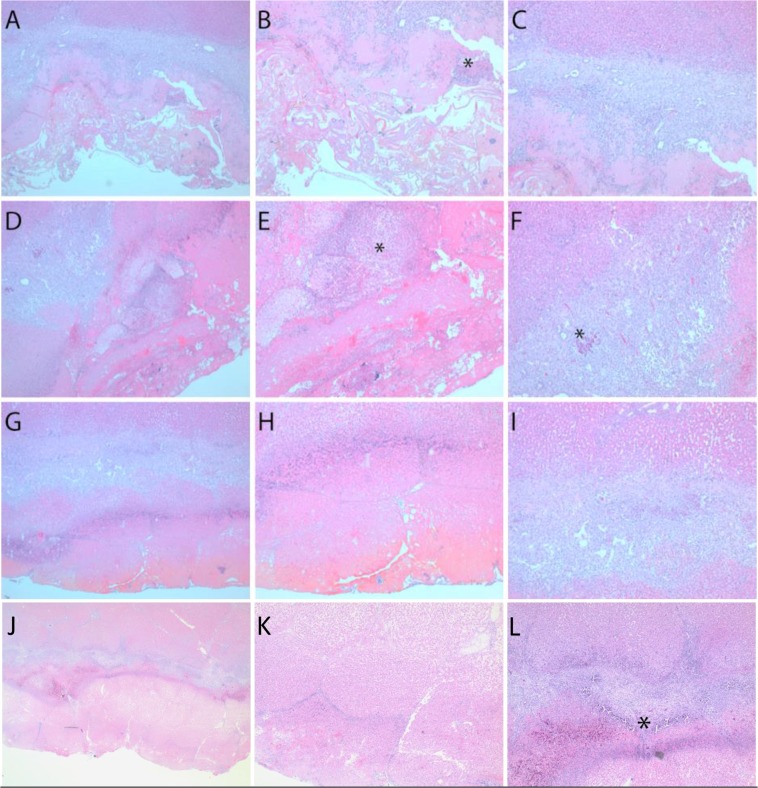
Histological changes in FBS-sealed (**A–C**) and CBS-sealed (**D–F**) liver resection planes compared with HPC (**G–I**) and CSC (**J–L**) groups. The left panel shows an overview. The central panel shows the surface in more detail. Fibrin (intense pink-stained material) is prominent in the CBS group (**E**), but also easily recognizable in the FBS group (**B**). There is a broad band of necrotic tissue in the HPC liver (**H**). Note, the presence of microabscesses (asterisks) in both sealant groups (**B,E**) and the blue rim-like demarcation line in the HPC liver. The right panel highlights changes at the liver interface (magnification: 50-fold). There is a small band of granulation tissue with scattered cholangioles and prominent hepatocellular regeneration as indicated by small cell changes in the adjacent parenchyma of the FBS-treated liver (**C**). In comparison, the granulation layer appears wider and more heavily inflamed in the CBS-treated liver (**F**), with focal calcification (asterisk) in the granulation tissue. Of note, the degree of hepatocellular regeneration is similar in both sealant groups. The foreign-body reaction is much less developed in the FBS-treated liver than the other groups. Histological changes at the liver resection plane of controls. (**J**) The overview reveals a broad band of necrotic liver tissue with hyperemia at the interface to the newly formed granulation tissue. (**K**) Medium power view demonstrating fibrin and hemorrhage at the outer part and necrotic liver parenchyma at the inner part of the wounded parenchyma. (**L**) There is a small rim of demarcation by polymorphic neutrophils, while the granulation tissue with focal calcifications (asterisk) separates the regenerating liver parenchyma from the resection surface.

The histopathological changes in the resection plane are illustrated in Figs 1 and 2. The rate of fibrin production was not significantly different between the HPC and CSC as well as FBS and CBS groups. As expected, more fibrin was detected in both sealant groups compared with the HPC (*p* < 0.001) and CSC (*p* < 0.001) groups. Significant differences were shown in parenchymal necrosis after liver resection between the sealant groups and HPC (*p* = 0.022) and CSC (*p* = 0.001) groups. Necrotic liver parenchyma at the resection plane was more abundant in the CSC group, and more necrosis was noted in the CBS group than in the FBS group. As expected, significantly less associated inflammatory reaction developed in the HPC group compared with the sealant groups (*p* = 0.009). However, there was no significant difference in inflammation between the CSC and sealant groups (*p* = 0.728). Although microabscess formation was less frequent in the HPC group than in both intervention groups, this difference did not reach statistical significance (*p* = 0.055). There was also no significant difference in microabscess formation between CSC, FBS, and CBS groups (*p* = 0.538). Similarly, the demarcation zone between the wound and the intact liver parenchyma was less developed in the HPC group compared with both sealant groups, but this difference was not statistically significant (*p* = 0.071). There were no significant differences in demarcation zone between the wound and the intact liver parenchyma between the CSC, FBS, and CBS groups (*p* = 0.161). Resection-plane-associated complete or confluent hemorrhage was lower in the sealant groups compared with the HPC (*p* = 0.049) and CSC groups (*p* = 0.035). The lowest rate of granulation tissue formation was seen in the HPC group (*p* = 0.014). Although not statistically significant, there was more granulation tissue formation in sealant groups than in the CSC group (*p* = 0.201). Additionally, both sealants increased the regeneration rate at the resection plane. The degree of hepatocellular regeneration was significantly higher in the sealant groups compared with the HPC group (*p* = 0.028) and CSC group (*p* = 0.033). Foreign-body reaction was present in all pigs in the CSC group (*p* = 0.161), but there were no significant differences in the presence and degree of foreign-body reaction between the HPC, FBS, and CBS groups (*p* = 0.343). However, a marked inter-individual difference in foreign-body reaction was noted (Fig. 2).

## Discussion

The liver is a highly vascularized organ. It has a sinusoidal architecture with specialized liver sinusoidal cells and does not contain a muscular layer, therefore vasoconstriction cannot be induced in the liver parenchyma after resection. Today, liver resection can be performed safely using different devices. However, resection-surface-related complications, such as posthepatectomy bile leakage and hemorrhage, remain challenging issues. The reported rates of posthepatectomy bile leakage and hemorrhage range between 0.4% and 12%^12,13^ and 1% and 8%^14,15^, respectively. Sealants have been used frequently to avoid these complications and to improve the postoperative outcome^6,7^.

Sealants effectively increase hemostasis at the resection surface during liver surgery^16^. Some studies have also shown less abdominal fluid collection and lower re-operation rates in sealant groups compared with control groups^17,18^. On the other hand, some authors reported no significant differences in resection-surface-related complications between patients treated with and without sealants^19,20^. Dressing materials are utilized worldwide but some centers avoid using them because of high costs and risk of inflammation^20,21^. The effectiveness of sealants remains controversial and their clinical relevance is not clear.

Sealants are foreign materials so may cause a foreign-body reaction, parenchymal inflammation, or provide a niche for microbial colonization after hepatectomy. However, the histopathological effects of different dressing materials on the resection plane after hepatectomy have not been reported in detail. To address this, we investigated histopathological changes to the liver resection surface after application of two different sealants (CBS and FBS). We used a swine model because the pig liver is similar in size and anatomy to the human liver and provides a greater scope for surgical evaluation compared with small animal models^22^. We compared the histopathological changes in sealant groups with two different control groups. To assess the pure effects of sealants on histopathological interactions with the liver surface after hepatectomy, we designed a HPC group, in which bleeding or bile leaks were not treated using standard clinical interventions (e.g., oversewing of the liver surface, argon beam coagulation, etc.). Additionally, to mirror the standard clinical practice, we designed a CSC group, in which we used the clamp-crushing method for transection, and controlled bleeding and bile leaks from the resection surface using oversewing of the liver surface and bipolar cautery.

We did not detect significant differences in microabscess formation and inflammatory demarcation between the wound and intact liver parenchyma, and in foreign-body reactions between both sealant groups and the HPC and CSC groups. The rate and severity of necrosis at the resection plane were significantly lower in the sealant groups than the CSC group. Hemorrhage was higher in the HPC group than in both sealant groups. The extent of hemorrhage in the sealant groups was also lower than in the CSC group. Inflammation at the resection plane was significantly more developed in both sealant groups than the HPC group. However, inflammation was not higher in the sealant groups than the CSC group. This is in line with findings from previous studies comparing the effects of FBS with other coagulation devices, including hot air coagulation and argon beam coagulation^9,11^. Katkhouda *et al*. reported significantly higher inflammation and significantly lower necrosis in an FBS group compared with an argon beam coagulation group^9^. Similarly, Tovar *et al*. observed lower necrosis and perilesional hemorrhage following the use of FBS sealants compared with hot air coagulation and control groups on postoperative days 5 and 10. Regeneration was complete on postoperative day 40 in the FBS group, but necrosis and perilesional fibrovascular tracts were still present in the other groups^11^.

We observed higher posthepatectomy granulation tissue formation in sealant groups compared with the HPC group. Although not statistically significant, a higher rate of moderate and prominent granulation tissue formation was also seen in both sealant groups compared with the CSC group. Hepatocellular regeneration in sealant groups was also higher compared with the HPC group and the CSC group. These findings suggest that sealants improve tissue repair and increase hepatocellular regeneration^21^. Posthepatectomy bile leakage can also influence liver regeneration. The rate of bilioma after liver resection was lower in pigs treated with sealants than control animals^17^. Suppressed liver function and impaired liver regeneration has also been demonstrated in patients^23^ and rats^24^ with posthepatectomy bile leakage.

There were some limitations to this study. First, the findings of present study do not fully mirror the standard clinical practice. Another limitation of our study was that we did not evaluate the correlation of these histopathological changes with long-term posthepatectomy clinical outcomes. Further studies are needed to investigate how the histopathological changes reported here associate with surgical complications such as posthepatectomy bile leakage and hemorrhage during longer term follow-ups.

In summary, sealants improve liver tissue repair and increase hepatocellular regeneration by enhancing the inflammatory reaction. However, sealants are expensive. In addition, different sealant materials (FBS and CBS) have different effects on histopathological changes to the liver surface. CBS increases hepatocellular necrosis and FBS increases the posthepatectomy hepatocellular regeneration rate. These differences and their correlations with long-term posthepatectomy clinical outcomes need further investigation. Future prospective randomized-controlled trials should be performed to establish the indications and criteria for sealant use in the clinical setting.

## Methods

To evaluate the pure histopathological effects and interactions of sealants on the liver surface after hepatectomy, we investigated two frequently used sealants in a standardized experimental swine model.

### Experimental groups

We used 36 landrace pigs (weight: 30.2 ± 2.1 kg), which were randomly allocated to a baseline HPC group (n = 9), FBS group (n = 9), CSC group (n = 9), or CBS group (n = 9).

### Anesthesia protocol

All operations and investigations were performed under general anesthesia. After premedication (azaperone 6 mg/kg intramuscularly [i.m.], ketamine 10 mg/kg i.m., and midazolamine hydrochloride 0.5 mg/kg i.m.), anesthesia was induced with ketamine (1 mg/kg intravenously [i.v.]), midazolamine hydrochloride (0.1 mg/kg i.v.), and atropine (0.04 mg/kg i.v.). During the operation, anesthesia was maintained with 1.5%–2% isoflurane. Ventilation was pressure-controlled in a half-closed system. The ventilation parameters were a tidal volume of 240 ml, frequency of 17/min, maximum pressure 83 of 24 cmH_2_O, and a positive end-expiratory pressure of 3–5 cmH_2_O. The pH, HCO_3_, pCO_2_, and pO_2_ concentrations were determined by routine analysis of arterial blood gases. The respiration parameters were then adapted to these values. During surgery, controlled infusion therapy was applied using Sterofundin (20 ml/kg/h). The body temperature was measured continuously using a rectal temperature probe and kept above 36 °C during experiments using a heated blanket.

### Hepatectomy

**Figure 3:**
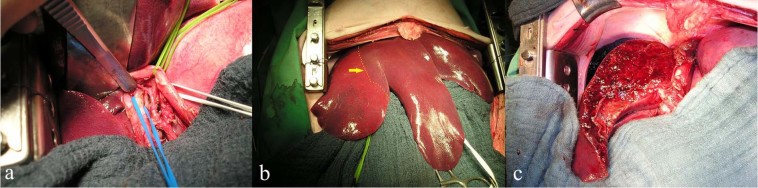
Hepatectomy phase (**a**) preparation of the left pedicle of the hepatic hilum. The left portal vein, hepatic artery, and bile duct were separated with blue, white, and yellow vascular loops, respectively (**b**) the demarcation zone (yellow arrow and line) (**c**) resected liver surface after left hemihepatectomy.

We performed an anatomical left hemihepatectomy (resection of left and medial hepatic segments) in all pigs. We used staplers (Endo GIA™ Universal Stapler, Covidien, Minneapolis, USA) for surgical transection in HPC, FBS, and CBS groups^25–28^, and the clamp-crushing method in the CSC group. As shown in Fig. 3a, the left pedicle of the hepatic hilum was prepared and ligated. After the demarcation line appeared (Fig. 3b), the demarcation zone was marked by electric cauterization to preserve the middle hepatic vein. To reduce blood loss during the hepatectomy phase, the central venous pressure was kept between 0 mmHg and 2 mmHg. Hepatectomy was performed using an Endo GIA stapler and 45 mm and 60 mm white magazines. No major bleeding from the portal and hepatic pedicles was observed. Bleeding and bile leaks from the resection surface were controlled by oversewing of the liver surface and bipolar cautery in the CSC group. To assess the pure effects of sealants on histopathological interactions with the liver surface, bleeding and bile leaks from the resection surface were not treated (e.g., with stitches, argon beam coagulation, etc.) in the HPC group or sealant groups (FBS or CBS) after hepatectomy (Fig. 3c). However, to trigger coagulation following liver resection, the liver surface was pressed with warm surgical sponges for 5 min in all animals.

### Protocols for sealant application

To seal the resected liver surface, two different commercial sealants were used in our animal model.

#### Tachosil^®^ (fibrinogen-based sealant)

TachoSil® (Takeda; Berlin, Germany) sealants are coated with coagulation factors (fibrinogen and thrombin) on their active surface. This active surface is marked yellow by riboflavin. Compared with prior products, TachoSil^®^ does not contain bovine aprotinin, which is a plasmin inhibitor. TachoSil^®^ was submerged in saline solution before application to the resected liver surface. The liver surface to be sealed was covered with TachoSil^®^ for 1 cm over the normal parenchymal surface. If more than one sealant was necessary, the sealant edges overlapped by more than 1 cm. Afterwards, TachoSil^®^ was slightly compressed to the resected hepatic surface for 3 min using moist gauze.

#### Tissufleece/tissucol Duo^®^ (collagen-based sealant/fibrin glue)

Tissufleece® (Baxter; Unterschleißheim, Germany) is a natural equine collagen and is an appropriate hematostatic material for covering the bleeding surface. It consists of heterologous collagen fibrils acquired from devitalized connective tissue and is fully reabsorbable. Tissucol Duo^®^ is a typical two component adhesive. The first part includes human plasma proteins (fibrinogen, factor XIII, plasma fibronectin, and the plasmin inhibitor aprotinin). The second part includes thrombin and CaCl_2_. Prior to application of Tissucol Duo^®^, injectors were kept for 20–30 min at room temperature for melting. The “treatment surface” was covered with a combination of the two injector contents and the TissuFleece^®^ boards were attached and left for 2 min. Two TissuFleece^®^ plates and 4 ml Tissucol Duo^®^ were applied to seal the hepatic resection field.

### Intraoperative evaluation

The heart rate was continuously monitored by surface electrocardiography (Hellige Monitoring Station, Germany). During the experiment, the systemic mean arterial pressure was continuously recorded from catheters inserted into the common carotid artery. Transhepatic flows including the hepatic arterial flow and portal vein flow were recorded. The relative bleeding time was defined as the time of starting the bleeding until the bleeding changed into oozing. The absolute bleeding time was defined as the time from the start of the bleeding until the bleeding completely stopped. Furthermore, to quantify the total blood loss (g), the dry weight of surgical sponges was preoperatively measured. To minimize the influence of fluids and other wound secretions, the liver was separated from other organs using plastic foil. Afterwards, when bleeding completely stopped, the weight of blood-soaked sponges was postoperatively measured. The total blood loss (g) was defined as the difference between the weight of blood-soaked sponges following the operation and the dry sponge weight measured before the operation^29^.

### Histopathological and laboratory evaluation

To investigate histopathological changes at the hepatic resection plane, a wedge biopsy was obtained 6 days following surgery. Tissue samples were fixed in 10% formaldehyde, embedded in paraffin, and sectioned for hematoxylin and eosin staining. The sections were evaluated by an experienced liver pathologist (T.L.), who was blinded to the experimental groups. The presence of microabscesses and a foreign-body reaction were qualitatively evaluated. The remaining parameters were scored semi-quantitatively after assessing the histological changes in the experimental groups. In particular, the width of the necrotic rim of liver parenchyma, the extent of fibrin exudation, and hemorrhage at the resection plane were evaluated. Inflammatory infiltrates at the surface (associated inflammation) were independently scored from the polymorphic granulocytes demarcating the resection plane. The degree of adjacent granulation tissue formation and inflammatory infiltrates present in the portal tracts of the more distant liver parenchyma were also evaluated. To evaluate hepatocellular regeneration, the presence and extent of small regenerating hepatocytes next to the granulation tissue were assessed. Table 2 gives an overview of the scoring system.

**Table 2:** Semi-quantitative scoring system to evaluate histopathological changes at the resection plane after hepatectomy.

Hepatocellular regeneration	Granulation tissue
0: not evident	0: not evident
1: mild	1: low
2: moderate	2: moderate
3: prominent	3: prominent
**Necrosis**	**Hemorrhage**
0: not evident	0: not evident
1: mild	1: focal
2: moderate	2: confluent
3: severe	3: complete resection plane
**Fibrin production**	**Microabscess formation**
0: not evident	0: not evident
1: mild	1: present
2: moderate	
3: prominent	
**Associated inflammation**	**Demarcation**
0: not evident	0: not evident
1: mild	1: focal
2: moderate	2: partial
3: high	3: complete

Laboratory assessments, including ALT, AST, total bilirubin, albumin levels, and INR, were performed preoperatively before hepatectomy and at the end of the experiments.

### Statistical analysis

Statistical analysis was performed using IBM SPSS Statistics for Windows, Version 22.0 (IBM Corp. Released 2013. Armonk, NY). Continuous data are expressed as mean values ± standard deviation and differences between groups were analyzed using the one-way ANOVA test. Categorical data were compared using the chi-square test of association. Histopathological data were analyzed using the Kruskal-Wallis test followed by the Bonferroni post-hoc method. Differences with a *p* value < 0.05 were accepted as statistically significant.

### Animal care

All animals received humane care according to institutional guidelines established for the Animal Care Facility at the University of Heidelberg during all experimental stages. Animals were housed and operated in the Interfacultary Biomedical Faculty at the University of Heidelberg. The study protocol was approved by the German Committee for Animal Care, Karlsruhe, Germany (AZ: 35-9185.81/G-45/12). At the end of experiments (day 6), all animals were sacrificed under deep anesthesia by intravenous injection of potassium chloride (2 mmol/kg).

## Data Availability

The datasets generated during and/or analysed during the current study are available from the corresponding author on reasonable request.
